# Supplementary material for: Exquisite Energy Savings at Cold Metal Forming of Threads through the Application of Polymers
Source: Polymers (Basel). 2022 Mar 8;14(6):1084. doi: 10.3390/polym14061084 (PMC8950108; doi:10.3390/polym14061084)
Supplement: Supplementary file 1 [file polymers-14-01084-s001.zip › polymers-1604591-supplementary.pdf]

Supporting Information to the Article

The exquisite energy savings at cold metal forming of threads through the application of polymers

Miroslav Píška[1], Petra Sliwková[1*], Zuzana Vnuková [1], Martin Petrenec[1] and Eva Sedláková-Valášková [1]

The statistical results of the deformation works, coeeficients of friction and specific forming forces for the tested polymers F,G,G+,H,I and G++ can be found in Figures S1-S6. First, the concentrations were prepared according to the producers or sellers, but some of them couldn´t be used because of their less powerful performance and rapid wear of the forming tap – e.g. Figure S1 for polymer F. The polymer G exhibited a very good effect on the deformation work – S2, but only at high concetrations what would make the industrial use very costly. The enriched polymer G+ showed a plateau effect in wide range of concentrations, however, the produced surface integrity showed many cracks and defects. The polymer H can be assessed as a very good competitor or an alternative to the polymer E, just the deformation works and the quality of the produced surfaces were in favour of the polymer E. On the analysis of the results used in the test the polymer I can´t be recommended in all studied parameters and catastrophic failure of many tools in the tests. The enriched polymer G++ didn´t confirmed our expectations and its price wouldn´t be possibly accepted in a similar industrial use like in the laboratory tests.

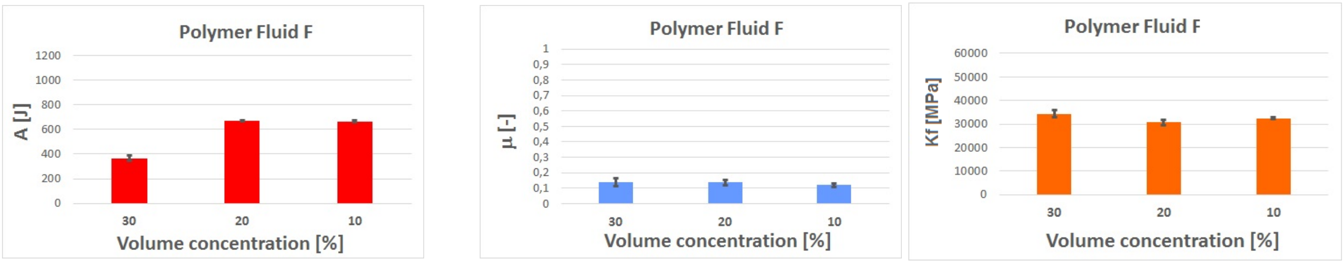

(**a**) (**b**) (**c**)

Figure S1. (**a**) Deformation works, (**b**) coefficient of friction and (**c**) specific forming force for the polymer F and various concentrations.

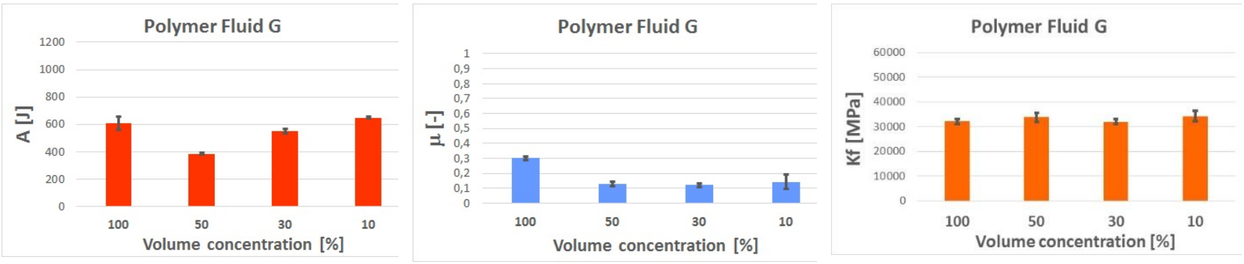

(**a**) (**b**) (**c**)

Figure S2. (**a**) Deformation works, (**b**) coefficient of friction and (**c**) specific forming force for the polymer G and various concentrations.

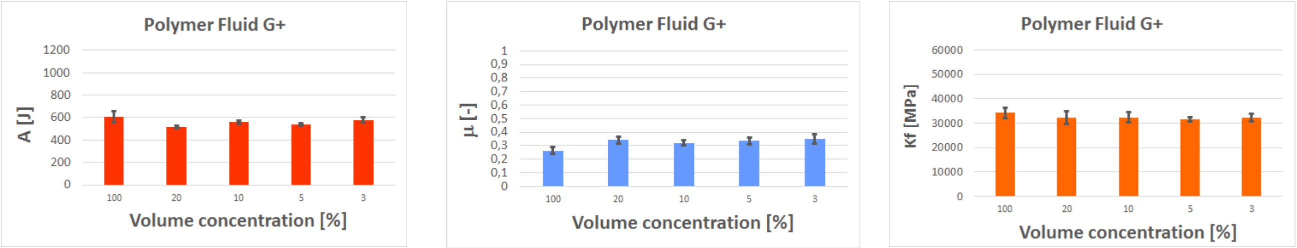

(a) (b) (c)

Figure S3. (**a**) Deformation works, (**b**) coefficient of friction and (**c**) specific forming force for the polymer G+ and various concentrations.

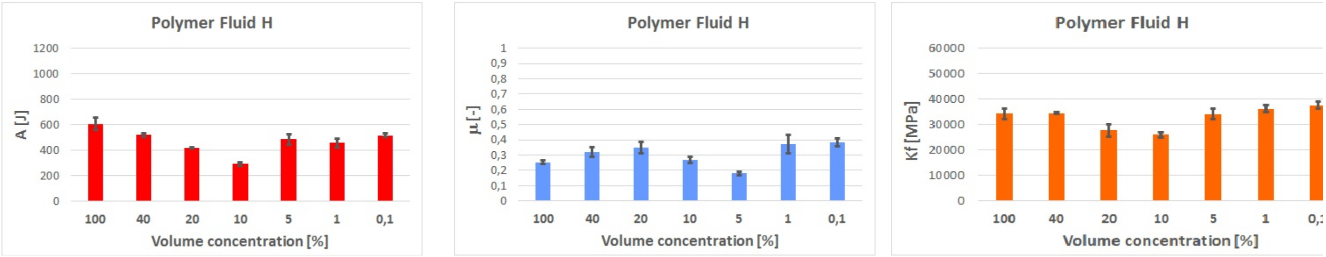

(a) (b) (c)

Figure S4. (**a**) Deformation works, (**b**) coefficient of friction and (**c**) specific forming force for the polymer H and various concentrations.

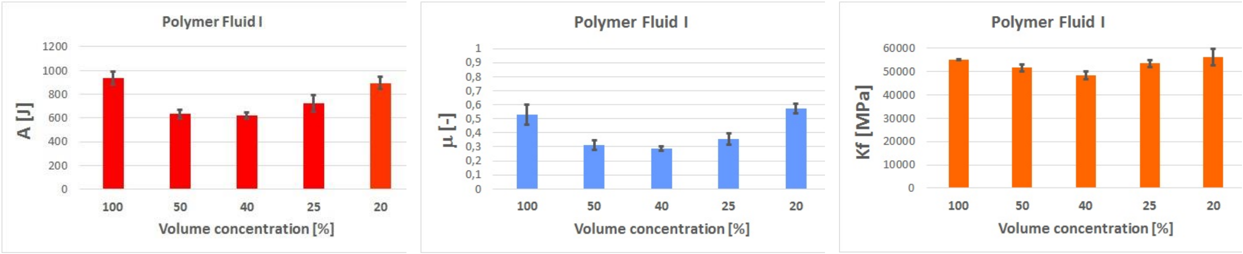

(a) b) (c)

Figure S5. (**a**) Deformation works, (**b**) coefficient of friction and (**c**) specific forming force for the polymer I and various concentrations.

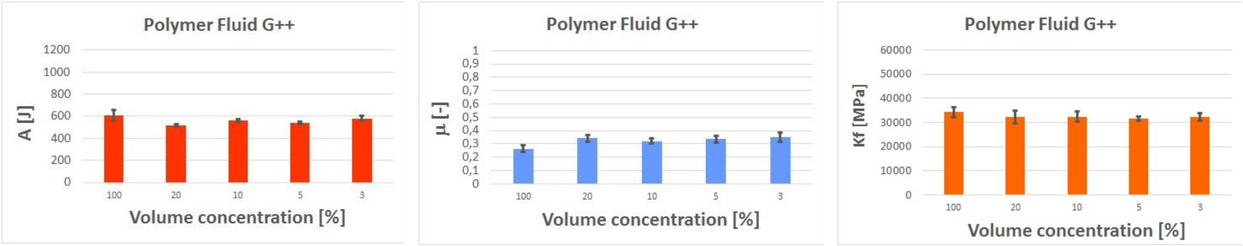

(a) b) (c)

Figure S6. (**a**) Deformation works, (**b**) coefficient of friction and (**c**) specific forming force for the enriched polymer G++ and various concentrations.
